# Subtractive Inhibition Assay for the Detection of *E. coli* O157:H7 Using Surface Plasmon Resonance

**DOI:** 10.3390/s110302728

**Published:** 2011-03-01

**Authors:** Yixian Wang, Zunzhong Ye, Chengyan Si, Yibin Ying

**Affiliations:** College of Biosystems Engineering and Food Science, Zhejiang University, Hangzhou 310029, Zhejiang, China; E-Mails:wang_yi_xian1986@sina.com (Y.W.); sichengyanzhj@163.com (C.S.); ybying@zju.edu.cn (Y.Y.)

**Keywords:** subtractive inhibition assay, SPR, *E. coli* O157:H7

## Abstract

A surface plasmon resonance (SPR) immunosensor was developed for the detection of *E. coli* O157:H7 by means of a new subtractive inhibition assay. In the subtractive inhibition assay, *E. coli* O157:H7 cells and goat polyclonal antibodies for *E. coli* O157:H7 were incubated for a short of time, and then the *E. coli* O157:H7 cells which bound antibodies were removed by a stepwise centrifugation process. The remaining free unbound antibodies were detected through interaction with rabbit anti-goat IgG polyclonal antibodies immobilized on the sensor chip using a BIAcore 3000 biosensor. The results showed that the signal was inversely correlated with the concentration of *E. coli* O157:H7 cells in a range from 3.0 × 10^4^ to 3.0 × 10^8^ cfu/mL with a detection limit of 3.0 × 10^4^ cfu/mL. Compared with direct SPR by immobilizing antibodies on the chip surface to capture the bacterial cells and ELISA for *E. coli* O157:H7 (detection limit: both 3.0 × 10^5^ cfu/mL in this paper), the detection limit of subtractive inhibition assay method was reduced by one order of magnitude. The method simplifies bacterial cell detection to protein-protein interaction, which has the potential for providing a practical alternative for the monitoring of *E. coli* O157:H7 and other pathogens.

## 1. Introduction

In recent times, *E. coli* O157:H7 as one of the major foodborne pathogenic bacteria and as such has attracted considerable attention. According to the U.S. Center for Disease Control and Prevention (CDC), outbreak data and the known ability of the organism to be passed from person to person in nursing homes, day-care centers, and other personal care facilities, indicate that the presence of as few as 10 *E. coli* O157:H7 could result in disease. It has been reported that there may be about 73,000 infections and 61 deaths occurring due to *E. coli* O157:H7 each year in the United States [1] and therefore it is of utmost importance to develop rapid and sensitive methods for *E. coli* O157:H7 detection.

By far, the most popular detection methods are culture and colony counting methods, polymerase chain reaction (PCR) and immunology-based methods and biosensors [2–6]. However, they are labor intensive and time consuming or professional operation limited. Biosensors, which incorporate a bioreceptor closely integrated with or connected to a transducer [7,8], have been proved to be a promising method for bacteria detection due to their portability, speed, sensitivity and possibility of on-the-spot detection [6,7,9], Surface plasmon resonance (SPR) biosensors are one kind of biosensor that has been widely used for bacterial detection [10–14]. A large number of direct SPR immunosensors have been used for the detection of bacterial cells by immobilizing antibodies directly on the chip surface to capture the bacterial cells [15–20]. Mazumdar immobilized antibodies on gold surface of each glass prism to capture *S. typhimurium* using the Plasmonic® SPR assay with a detection limit of 1.25 × 10^5^ cfu/mL [20]. Subramanian reported that the detection limit of direct surface plasmon resonance assay for *Escherichia coli* O157:H7 detection was 10^6^ cfu/mL [17]. The method based on the surface capture of cells has some limits to reduce the sensitivity of immunosensors [21–24]. Firstly, the effective penetration depth of the evanescent field which arises under conditions of total internal reflection is approximately 300 nm. It means that only refractive index changes occurring within the 300 nm distance from the surface will cause a change in the generated SPR signal. Bacteria such as *E. coli* O157:H7 with size of about 1 μm probably only interact with the top of the dextran layer that coats the gold surface and therefore only a small portion of the cell which is in close contact with the sensor surface will produce a measurable signal, which decreases the sensitivity of SPR for *E. coli* O157:H7 detection [21–24]. In addition, due to the large size of bacterial cells, direct cell binding requires that the cell-antibody binding affinity must be high to withstand the effect of shear force created by the laminar flow in the microflow channels [24]. Finally, Biacore instruments average the SPR angle over an area of approximately 0.25 mm^2^ on the sensor surface. As the sizes of bacterial cells are large, they will not evenly cover the area measured due to steric hindrance, which will decrease the signal response [24].

**Figure 1.**
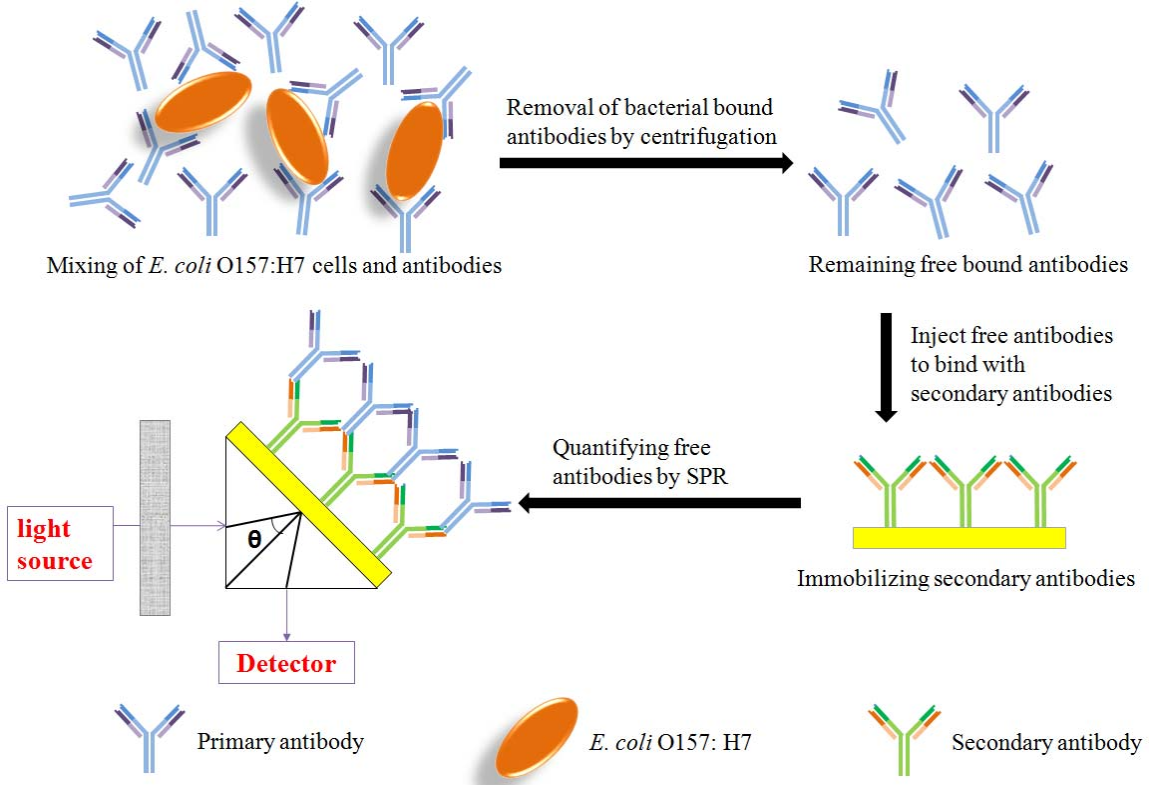
Principle of a subtractive inhibition assay (SIA) using SPR.

In this paper, to avoid the defects of SPR detection due to the size of bacteria, a new subtractive inhibition assay using SPR detection of *E. coli* O157:H7 was developed. In the proposed assay, *E. coli* O157:H7 cells and antibodies were incubated for a short of time, and the *E. coli* O157:H7 cells which bound antibodies were removed by a stepwise centrifugation process. Then the remaining free unbound antibodies were quantified through binding with anti-antibody immobilized on the sensor chip using BIAcore 3000 biosensor, which were inversely proportional to the *E. coli* O157:H7 cell concentration (Figure 1). This method simplifies bacterial cells detection to protein-protein interaction, which increases sensitivity of SPR for *E. coli* O157:H7 detection.

## 2. Experimental Section

### 2.1. Regents

The reagents were obtained from the following sources: goat polyclonal antibody for *E. coli* O157:H7 and *E. coli* O157:H7 positive control were from KPL (Gaithersburg, MD, USA); *E. coli* DH5α (ATCC PTA-3137) was obtained from College of Food Science at Zhejiang University; Rabbit anti-goat IgG polyclonal antibody was from Boster (Wuhan, China); Sensor chip CM5, HBS running buffer (10 mM HEPES, 150 mM NaCl, 3.8 mM EDTA, 0.05% (v/v) Tween), 10 mM acetate buffer (pH 4.5) and 1 M ethanolamine (pH 8.5) were from GE Healthcare Bio-Sciences AB (Uppsala, Sweden); Phosphate buffered saline (PBS, pH 7.4), *N*-hydroxysuccinimide (NHS) and *N*-ethyl-*N*-(dimethylaminopropyl) carbodiimide hydrochloride (EDC) were from Sigma (St. Louis, MO, USA). All other regents were all chemical analytical grades.

### 2.2. ELISA for E. coli O157:H7 Detection

Microtiter plates were coated with 100 μL of 10 μg/mL of goat polyclonal antibodies for *E. coli* O157:H7 in phosphate buffered saline (PBS) overnight at 4 °C, and then washed three times with PBS + 0.05% Tween-20 (PBST). 300 μL 3% bovine serum albumin (BSA) was used to block unbound sites each plate for 30 min, followed washing with PBST for three times. One hundred μL of *E. coli* O157:H7 samples of different concentration from 3.0 × 10^2^–3.0 × 10^8^ cfu/mL were loaded onto each plate and incubated for 1 h at room temperature. The plates were washed three times with PBST and then 100 μL 1,000-fold decreasing dilutions of peroxidase-conjugated affinipure donkey anti-goat IgG (H+L) was added to each well. Following 30 min incubation and a final wash step, the plates were developed with TMB, stopped after 10 min with H_2_SO_4_ and read at a wavelength of 450 nm on a spectrophotometer.

### 2.3. Direct SPR by Immobilizing Antibodies on the Chip Surface to Capture the Bacterial Cells

After activation by injecting 70 μL mixture of 0.1 M NHS with 0.4 M EDC at 10 μL/min for 7 min, a 100 μg/mL goat polyclonal antibody for *E. coli* O157:H7 in 10 mM acetate buffer, pH 4.5, was injected over the chip surface at 10 μL/min for 10 min. Unreacted sites were subsequently deactivated by injecting 1 M ethanolamine, pH 8.5, at 10 μL/min for 7 min. Then, different concentration of diluted *E. coli* O157:H7 (3.0 × 10^1^–3.0 × 10^8^ cfu/mL) in running buffer were flowed for 5 min at 10 μL/min for direct detection. After the bacterial cells binding, the chip surface was regenerated by injecting 10 μL of 15 mM NaOH every time.

### 2.4. Subtractive Inhibition Assay

#### 2.4.1. Free Antibody Centrifugation Separation

Three hundred μL of a 50 μg/mL anti-*E. coli* O157:H7 polyclonal antibody solution diluted in PBS, pH 7.4, was mixed with 300 μL of *E. coli* O157:H7 with series of concentration from 10^1^–10^8^ cfu/mL. The final anti-*E. coli* O157:H7 polyclonal antibody concentration was 25 μg/mL. Each mixture was incubated in rotating for 1 h at room temperature, followed stepwise centrifugation process for 2 min intervals at 50, 200, 400, 800, 1,200, 1,800 and 3,200 × g to separate the cells bound antibodies from the remaining free unbound antibodies. Get the remaining free unbound antibodies by drawing 500 μL supernatant fluids from the centrifugal tube.

#### 2.4.2. Antibody Immobilization and Assay Setup

The remaining free unbound antibodies were quantified using a BIAcore 3000TM instrument. The CM 5 sensor chip was activated by injecting 70 μL mixture of 0.1 M NHS with 0.4 M EDC at 10 μL/min for 7 min. A 100 μg/mL rabbit anti-goat (Fab portion) polyclonal antibodies in 10 mM acetate buffer, pH 4.5, were injected over the chip surface at 10 μL/min for 10 min. Unreacted sites were subsequently deactivated by injecting 1 M ethanolamine, pH 8.5, at 10 μL/min for 7 min. Then the remaining free unbound antibodies were injected in the chip surface to bind with the secondary antibodies. After the antibodies binding, the chip surface was regenerated by injecting 10 μL of 15 mM NaOH every time.

### 2.5. Specificity Testing

The specificity of the biosensor was confirmed by detecting *E. coli* DH5α, one of *E. coli* serotypes. 300 μL 3.0 × 10^8^ cfu/mL *E. coli* DH5α mixed with 300 μL of a 50 μg/mL anti-*E. coli* O157:H7 polyclonal antibody solution. And then the mixture was with the same free antibody centrifugation separation and detection method as *E. coli* O157:H7 detection.

## 3. Results and Discussion

### 3.1. ELISA for Validity of Antibody for E. coli O157:H7 Detection

**Figure 2.**
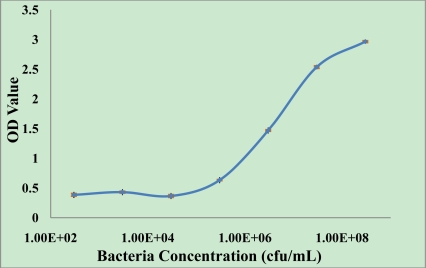
Detection of *E. coli* O157:H7 based on ELISA.

ELISA analysis was used as a conventional immunoassay method for comparison with the biosensor method, and to observe the quality of the *E. coli* O157:H7 antibodies used in the immunosensor. Eight concentrations of *E. coli* O157:H7 (3.0 × 10^1^ to 3.0 × 10^8^ cfu/mL) were assayed with the ELISA, and the results are shown in Figure 2. From 3.0 × 10^5^ cfu/mL, increasing the bacteria concentration resulted in an increase of OD value (2.9647, 2.5363, 1.4685 and 0.6323 observed with 3.0 × 10^8^, 3.0 × 10^7^, 3.0 × 10^6^, 3.0 × 10^5^ cfu/mL *E. coli* O157:H7 cells respectively). And the standard deviation (SD) for each point of the standard curves (3.0 × 10^8^, 3.0 × 10^7^, 3.0 × 10^6^, 3.0 × 10^5^ cfu/mL and control) were 1.5%, 2.0%, 1.2%, 0.9%, 2.2%, respectively. The results indicated that the purchased anti-*E. coli* O157:H7 antibody allowed the detection of *E. coli* O157:H7. The detection limit of this ELISA was 3.0 × 10^5^ cfu/mL of *E. coli* O157:H7.

### 3.2. Direct SPR Immobilizing Antibodies on the Chip Surface to Capture the Bacterial Cells

The first step of the direct assay was the immobilization of the capture antibodies on the CM5 chip surface, and a significant increase in the sensorgram signal (RU = 17,000) was observed, indicating a stable binding interaction between the CM5 chip surface and the captured antibody. The incubation time was only 10 min. Through immobilizing antibodies directly on the chip surface to capture the bacterial cells, a detectable change in RU due to its binding to the bacterial cells was obtained only at and above a cell concentration of 3.0 × 10^5^ cfu/mL. 47, 76, 160 and 288 RU observed from binding with 3.0 × 10^5^, 3.0 × 10^6^, 3.0 × 10^7^, 3.0 × 10^8^ cfu/mL *E. coli* O157:H7 cells, respectively. The standard deviation (SD) for each point of the standard curves (3.0 × 10^8^, 3.0 × 10^7^, 3.0 × 10^6^, 3.0 × 10^5^ cfu/mL and control) were 8.1%, 2.4%, 4.7%, 3.3%, 3.1%, respectively. The lower limit of detection (LLD) is defined as the concentration of cells resulting in a detection signal that is the average value of the detection signal obtained due to control plus 3 times the standard deviation [20]. The detection range was between 3.0 × 10^5^ and 3.0 × 10^8^ cfu/mL with a limit of detection of 3.0 × 10^5^ cfu/mL.

### 3.3. Centrifugation Effect

Haines *et al.* have reported removal of the cells-bound antibodies by a method such as filtration, however, that method was not suitable for complex and viscous matrices [25]. It has been reported that separate the remaining free antibodies from the bacterial cells-bound antibodies by centrifugation for 1 min intervals at 50, 200, 450, 800, 1,200, 1,800 and 3,200 × g was possible [23]. Therefore, in this paper, a stepwise centrifugation process has been applied to separate the remaining free unbound antibodies from the bacterial cells that bound antibodies. But according to the above method, the signal of 10^8^ cfu/mL was larger than the signal of 3.0 × 10^7^ cfu/mL, possibly because the sizes of *E. coli* O157:H7 cells (about 1 μm) are smaller than *Listeria monocytogenes* cells, resulting in the need for greater centrifugal force and longer centrifugation times, so we increased the centrifugation time by centrifuging for 2 min intervals at 50, 200, 400, 800, 1,200, 1,800 and 3,200 × g, confirming the method’s principle, resulting in a decreasing signal with increasing *E. coli* O157:H7 bacteria cell concentration. Optimization of the centrifugation time according to the size of *E. coli* O157:H7 cells was the key step for the success of this experiment.

### 3.4. Sensor Chip Preparation

**Figure 3.**
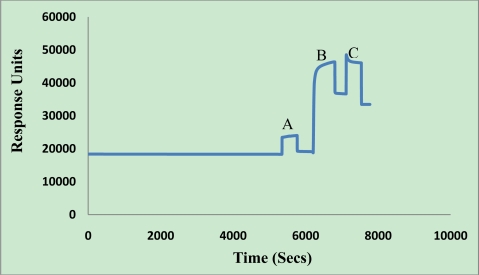
Plot of sensor chip preparation: **(A)** activation. **(B)** rabbit anti-goat IgG polyclonal antibodies immobilization. **(C)** ethanolamine blocking.

The subtractive inhibition assay was further implemented into a Biacore® 3000 SPR sensor. The scheme for the sensor setup used in this work is shown in Figure 1. The dextran layer was immobilized on the gold surface of CM5 chip beforehand. Then the surface was activated with an NHS-EDC solution, washed with running buffer, and a goat anti-mouse IgG polyclonal antibody was immobilized to a CM5 chip surface (17,556 RU) through an amide bond, as shown in Figure 3. The binding response was compared to that of a reference channel chip surface (same activation using EDC and NHS, blocking using ethanolamine, but without immobilizing secondary antibody), which gave insignificant binding, thereby illustrating the specificity of the binding response (data not shown).

### 3.5. Surface Regeneration

The rabbit anti-goat IgG polyclonal antibodies were immobilized on the CM5 chip and optimal regeneration conditions were investigated. The regeneration solution should remove the remaining free antibodies from the anti-goat IgG polyclonal antibodies without affecting the activity of the secondary antibodies. The regeneration solution of 10 μL 15 mM NaOH at a flow rate of 10 μL/min was sufficient for surface regeneration. The surface activity of the chip decreased 6.0% by repeated 50 binding and regeneration cycles, which proved the excellent long-term surface performance.

### 3.6. Sensor Performance

**Figure 4.**
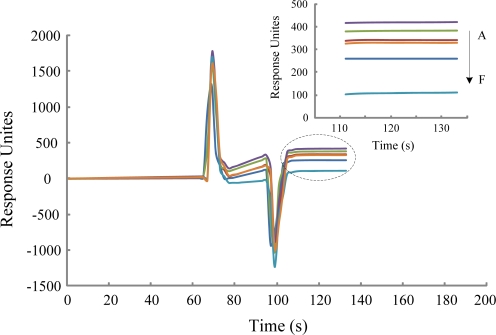
Overlay plot from one assay demonstrating that the binding response is inversely proportional to the *E. coli* O157:H7 concentration: **(A)** control sample. **(B)** 3.0 × 104 cfu/mL. **(C)** 3.0 × 10^5^ cfu/mL. **(D)** 3.0 × 10^6^ cfu/mL. **(E)** 3.0 × 10^7^ cfu/mL. **(F)** 3.0 × 10^8^ cfu/mL.

In the subtractive inhibition assay, different concentrations of *E. coli* O157:H7 cells and antibodies were incubated and then the *E. coli* O157:H7 cells which bound antibody were removed by a stepwise centrifugation process. The samples of free unbound antibodies were quantified in duplicate out of order through binding with anti-antibody immobilized on the sensor chip using BIAcore 3000 biosensor. As expected, and as shown in Figure 4, the binding responses were inversely proportional to the concentration of the *E. coli* O157:H7 cells, which verified the subtractive inhibition assay principle. 166, 285, 373, 389, 419 and 449 RU observed from supernatants from samples incubated with 3.0 × 10^8^, 3.0 × 10^7^, 3.0 × 10^6^, 3.0 × 10^5^, 3.0 × 10^4^ and 0 cfu/mL *E. coli* O157:H7 cells, respectively.

**Figure 5.**
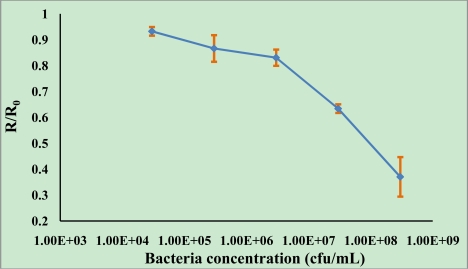
The plot between SPR response and bacterial cells concentration.

The resonance dips of different bacterial cells concentration from 3.0 × 10^8^ to 3.0 × 10^4^ were 283, 164, 76, 60 and 30, respectively. The signal was correlated with the concentration of *E. coli* O157:H7 cells in a range from 3.0 × 10^8^ to 3.0 × 10^4^ cfu/mL. Normalized data (R/R_0_) was obtained with the average response for each sample(R) divided by the average of the control (sample without bacterial cells, R_0_). A calibration curve using normalized data (R/R_0_) plotted against the *E. coli* O157:H7 cells concentrations, was constructed (Figure 5). According to Figure 5, the range of detection was found to be approximately 3.0 × 10^4^–3.0 × 10^8^ cfu/mL. The standard deviations for each point of the standard curves (3.0 × 10^8^, 3.0 × 10^7^, 3.0 × 10^6^, 3.0 × 10^5^, 3.0 × 10^4^ cfu/mL) were 7.6%, 1.7%, 3.1%, 5.1%, 1.7%, respectively, which illustrates the good reproducibility of the immunoassay.

In the subtractive inhibition assay, the lower limit of detection (LLD) is defined as the concentration of cells resulting in a detection signal that is the average value of the detection signal obtained due to control minus three times the standard deviation. The relative standard deviation for control samples was 3.5%. Therefore, the detection limit of SPR detection based on subtractive inhibition assay is 3.0 × 10^4^ cfu/mL, which is one order of magnitude less compared with direct SPR detection immobilizing antibodies on the chip surface to capture the bacterial cells and ELISA method for *E. coli* O157:H7 detection (the detection limit: both 3.0 × 10^5^ cfu/mL). That method is of significance because there have been problems encountered in the use of SPR for bacterial cell detection. It simplifies bacterial cells detection to protein-protein interaction, which avoid the defects of SPR detection due to the size of bacteria, including the cell size exceeding the 300 nm range of the evanescent field wave, high fluid force acting on captured cells, and limited mass transfer. Therefore the subtractive inhibition assay has improved the sensitivity of the sensors for *E. coli* O157:H7 detection.

The traditional culture and colony counting method has been a practical method for the detection and identification of *E. coli* O157:H7 in food, including microbiological culturing and isolation of the pathogen, followed by confirmation by biochemical and serological tests, which takes up to 5–7 days to get a confirmed result [26]. Although it can provide reliable results, it is time consuming, which is not suitable for rapid assay for *E. coli* O157:H7 and other pathogens in the food industry. The polymerase chain reaction (PCR) and enzyme-linked immunosorbent assay (ELISA) are a lot less time-consuming than the traditional culture and colony counting methods, and usually take 4–5 hours to produce detection result [6,27]. Compared with above mentioned methods for *E. coli* O157:H7 detection, the subtractive inhibition assay based on SPR, excluding sample incubation, centrifugation, secondary antibodies immobilization and interaction time, was less than 2 h. It has reduced the testing time effectively and can detect multiple samples in succession with the process of regeneration, which have the potential for providing a practical alternative for the monitoring of *E. coli* O157:H7 and other pathogens in the food industry.

### 3.7. Specificity of the Biosensor

**Figure 6.**
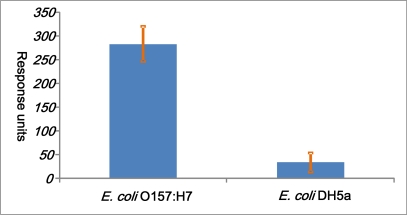
The cross-reactivity studies plot between *E. coli* O157:H7 and *E. coli* DH5α (3.0 × 10^8^).

The specificity of the biosensor was confirmed by detecting *E. coli* DH5α, one of *E. coli* serotypes. For *E. coli* DH5α (the concentration of 3.0 × 10^8^ cfu/mL), the response signal was 412, while the response signal *of E. coli* O157:H7 cells (3.0 × 10^8^ cfu/mL) was 166. The *E. coli* O157:H7 gave a binding response of 283 and weak reactivity was found with *E. coli* DH5α with a signal dip of 37 (Figure 6). This suggests that *E. coli* DH5α did not have strong binding force with anti-*E. coli* O157:H7 polyclonal antibodies as *E. coli* O157:H7, demonstrating specificity of the biosensor in the presence of non-target bacterial cells.

## 4. Conclusions

A new subtractive inhibition assay using SPR detection of *E. coli* O157:H7 has been clearly established. Unlike the direct SPR for bacterial detection by immobilizing the antibody on the chip surface, in the subtractive inhibition assay, the remaining free unbound antibodies obtained by stepwise centrifugation process after *E. coli* O157:H7 cells and relevant antibodies incubation, were detected through interaction with secondary antibody immobilized on the sensor chip using a BIAcore 3000 biosensor. The results showed that the signal was inversely correlated with the concentration of *E. coli* O157:H7 cells in a range from 3.0 × 10^4^ to 3.0 × 10^8^ cfu/mL. The detection limit was 3.0 × 10^4^ cfu/mL, compared with direct SPR by immobilizing antibodies on the chip surface to capture the bacterial cells and ELISA methods with the same bacterial cells and relevant antibodies (detection limit: 3.0 × 10^5^ cfu/mL in this paper), the detection limit of subtractive inhibition assay method was reduced by one order of magnitude. The subtractive inhibition assay method simplified bacterial cell detection to protein-protein interaction, and can increase the sensitivity of SPR biosensors for *E. coli* O157:H7 detection. The assay time required for sample detection was less than 2 h and the sample requirement for each analysis was only 5 μL, which reduced the testing time and sample volume effectively compared with the traditional methods for bacterial detection. The present assay can be used in automated mode with the ability to rapidly analyze a large number of samples and has specificity in the presence of non-target bacterial cells.

The subtractive inhibition assay method based on SPR can also be applied to the detection of other organisms suffering from oversize effects, such as virus, bacteria, fungal, cells and so on. The simplicity of this SPR-based subtractive inhibition assay for detection of *E. coli* O157:H7 clearly demonstrates the potential of the SPR technique to be used as a rapid detection tool for food microbiological safety.
